# Frailty index and risk of delirium in hospitalized patients: a two-sample Mendelian randomization study

**DOI:** 10.3389/fmed.2024.1361437

**Published:** 2024-05-22

**Authors:** Yu Chen, Fang Feng, Qun Li, Hong Guo, Lu Zhang, Jian Liu

**Affiliations:** ^1^The First School of Clinical Medicine of Lanzhou University, Lanzhou, Gansu, China; ^2^Intensive Care Unit, Lanzhou University Second Hospital, Lanzhou, Gansu, China; ^3^Intensive Care Unit, The First School of Clinical Medicine of Lanzhou University, Lanzhou, Gansu, China; ^4^Intensive Care Unit, Gansu Provincial Central Hospital, Lanzhou, Gansu, China

**Keywords:** frailty index, delirium, Mendelian randomization study, intensive care, nursing care

## Abstract

**Objective:**

Observational studies suggest that the frailty index (FI) is closely related to delirium, but the relationship between them is still uncertain due to the influence of various confounding factors. Therefore, two-sample Mendelian randomization (MR) was used to explore the causal relationship between the FI and delirium risk.

**Methods:**

This study obtained pooled statistics for the FI and delirium from two of the most extensive genome-wide association studies. To make the results more robust and reliable, supplementary analyses were performed using several robust analytical methods (inverse-variance weighting, MR-Egger regression, and weighted median). In addition, this study used the MR-Egger intercept test, Cochran’s Q test, funnel plots and the leave-one-out method to evaluate the pleiotropy and heterogeneity among the abovementioned genetic variation instrumental variables.

**Results:**

Frailty might increase the relative risk of delirium, as shown by IVW (OR = 1.849, 95% CI 0.027∼2.067, *P* = 0.044), weighted median (OR = 1.726, 95% CI −0.178∼2.664, *P* = 0.083), MR-Egger regression (OR = 1.768, 95% CI −3.08∼6.171, *P* = 0.525) and leave-one-out sensitivity analysis (*P* = 0.058). Although the WME method and MR–Egger regression analysis showed no statistically significant causal relationship between the FI and the risk of delirium, the direction of the causal effect was consistent with the IVW method.

**Conclusion:**

There is a notable correlation between a higher FI and an elevated risk of delirium. This indicates that healthcare providers should take proactive measures to prevent delirium in hospitalized patients with a higher FI.

## Relevance statement

With the arrival of an aging society, frailty has received increasing attention in clinical nursing work, and previous observational studies have shown that frailty can increase the incidence of delirium, leading to poor prognosis. Both frailty and delirium are complications that require attention in daily nursing work. Therefore, this study intends to use Mendelian randomization method to explore the causal relationship between frailty and delirium, thereby providing a theory for improving the quality of clinical nursing care.

## 1 Background

Delirium, a profound and sudden-onset neuropsychiatric condition, is distinguished by compromised focus and awareness, an ever-changing trajectory, and general cognitive decline (1). Greatly prevalent among the elderly and critically ill, this syndrome has numerous predisposing factors and frequently manifests as a complication of sudden illness, substance overconsumption or cessation, surgical intervention, or disturbances in electrolyte or metabolic levels; it can even arise solely due to hospitalization (2). Despite the high frequency of delirium cases, its identification, diagnosis, and treatment in clinical practice are often neglected, misjudged, or inadequately handled.

Observational studies conducted in Italy aimed to examine the potential association between frailty and delirium in hospitalized older adults and in different contexts (3), and others were investigated the impact of these syndromes on outcomes including maintaining attention, functional status and mortality (4, 5). Different researchers also conducted systematic reviews and meta-analyses of the literature to address this issue, showing that frailty and delirium are common in geriatric practice, but their association with each other and their effect on short-term mortality in the general population hospitalized for acute conditions remains largely unknown (6, 7). The findings of this study suggest that frailty is indeed associated with delirium in hospitalized elderly patients. Furthermore, the presence of both conditions, either alone or in combination, leads to increased short-term mortality rates.

Notably, the connection between frailty and delirium is frequently assessed through observation-based studies. The currently debated aspect of the frailty-delirium relationship stems from the biases, confounding factors, and constraints inherent in observational studies. The utilization of Mendelian randomization (MR) provides a robust method for identifying causal links between risk factors and diseases by leveraging genetic variation as an instrumental variable (8). Given that genetic variation is determined at conception, it is generally less susceptible to external factors. Additionally, MR research mitigates concerns surrounding the causal sequence, a critical aspect of causal inference and the foundation of establishing causality (9). Hence, this study incorporates a two-sample MR analysis utilizing publicly available data from genome-wide association studies (GWAS) to investigate the causal association between the FI and the susceptibility to delirium.

## 2 Materials and methods

### 2.1 Design

To assess whether there is a causal relationship between frailty and delirium, a two-sample MR was used in this study.

### 2.2 Data sources

For the purpose of this analysis, single-nucleotide polymorphisms (SNPs) associated with the FI were obtained from the European Bioinformatics Institute (EBI) as instrumental variables. The working variable (ID: ebi-a-GCST90020053) originates from a Genome-wide association study (GWAS) on frailty (10), which included 164,610 samples of individuals aged 60 to 70 years. The sample population consisted of 84,819 females (51.3%), the detail information is in the Supplementary Table 1. The relevant SNP data on delirium was obtained from the Finnish database. This dataset mainly comes from the European population, contains a total of 16,380,452 SNPs and the inclusion criteria were acute or subacute: (1) brain syndrome; (2) confusional state (nonalcoholic); (3) infective psychosis; (4) organic reaction; (5) psycho-organic syndrome. Delirium cases were excluded if they were described as delirium tremens, alcohol-induced, or unspecified. Delirium was diagnosed based on the patient’s ICD-10 code at discharge, and the database did not specify which delirium assessment scale was used to assess the patient during the hospitalization. And the detail basal information of delirium datasets is in the Supplementary Table 2 and Supplementary Figures 1, 2.

### 2.3 Selection of instrumental variables

First, this study used Plink software to screen out SNPs with *P* < 5 × 10^–8^, a genetic distance of 10,000 kb and linkage disequilibrium (LD) *r*^2^ < 0.001 from the FI database (11). Second, the catalog and PhenoScanner databases were also used to further verify whether the above included SNPs were related to other confounding factors (12). Finally, the F statistic was used to evaluate whether the included SNPs were affected by weak instrumental variables (13). If the F statistic of SNPs is less than 10, it indicates that there is a possibility of weak instrumental variable bias in the SNPs, and then it will be eliminated to avoid affecting the results.

### 2.4 Statistics

This study mainly used the inverse-variance weighted (IVW) method, wherein the existence of the intercept item is not considered in the regression and the reciprocal of the outcome variance is used as the weight for fitting (14). Among the different IVW approaches, the IVW fixed-effects model was mainly used in the absence of any underlying heterogeneity of horizontal pleiotropic effects. If heterogeneity existed, a random-effects model was used. Second, this study used methods such as MR-Egger regression and the weighted median estimator (WME) to further supplement the above conclusions. The biggest difference between the MR-Egger method and the IVW method is that the existence of the intercept item is considered in the MR–Egger regression, and it also uses the reciprocal of the outcome variance as the weight for fitting (15). The weighted median method was defined as the median of the weighted empirical density function of ratio estimates, from which causality was assessed if at least 50% of the information in the analysis came from valid tools.

### 2.5 Analyses of horizontal pleiotropy and heterogeneity

This study used the “leave-one-out” sensitivity analysis by removing individual SNPs one-at-a-time to assess whether that variation drove the association between exposure and outcome variables. Second, to clarify whether there was horizontal pleiotropy in the MR analysis, this study also carried out MR-Egger intercept detection. If the intercept item in the MR–Egger intercept analysis had obvious statistical significance, then it indicated that the study had obvious significance. Finally, this study also used Cochran’s Q statistic to detect heterogeneity. Significant heterogeneity in the results of the analysis was demonstrated if the Cochran’s Q test result was statistically significant. *P* < 0.05 was considered to indicate statistical significance. All statistical analyses were performed using R program (version 4.2.0), including packages such as TwoSampleMR.

## 3 Results

### 3.1 Selection of instrumental variables

In this study, 14 genome-wide significant SNPs closely related to frailty were selected as instrumental variables (see Table 1). The included SNPs explained approximately 7.41% of the phenotypic variation, and the F values of the included SNPs were all greater than 10, which proved that the study was not easily affected by weak instrumental variables. The MR-Egger regression intercept and leave-one-out sensitivity analysis showed no horizontal pleiotropy for any instrumental variable (see Table 2).

**TABLE 1:** Basic information of each SNP in the FI and delirium databases.

SNP	Chr	EA	OA	EAF	FI			Delirium		
					**Beta**	**SE**	***P*-value**	**Beta**	**SE**	***P*-value**
rs10891490	11	C	T	0.5915	−0.0188	0.0034	2.00E-08	−.0369	0.042	0.3795
rs12739243	1	C	T	0.2206	−0.0242	0.004	1.28E-09	−0.07	0.044	0.1113
rs1363103	5	C	T	0.38	−0.0191	0.0034	2.23E-08	0.0115	0.0413	0.781601
rs17612102	15	C	T	0.5933	0.0187	0.0034	2.85E-08	−0.0103	0.0408	0.7997
rs2071207	3	C	T	0.478	−0.0187	0.0033	1.47E-08	−0.0832	0.0403	0.03886
rs2396766	7	A	G	0.4725	0.0201	0.0033	1.22E-09	−0.0444	0.0403	0.2708
rs3959554	15	G	A	0.4177	0.0189	0.0034	1.74E-08	0.0406	0.0431	0.3457
rs4146140	10	T	C	0.3811	−0.0198	0.0034	6.83E-09	0.0161	0.0427	0.7065
rs4952693	2	T	C	0.3734	−0.0194	0.0034	1.47E-08	−0.0241	0.0409	0.555699
rs56299474	8	A	C	0.1733	0.0241	0.0044	3.94E-08	−0.0235	0.0548	0.667599
rs583514	3	C	T	0.5111	0.0199	0.0033	1.65E-09	0.0609	0.0405	0.1321
rs8089807	18	T	C	0.1866	−0.0248	0.0043	6.50E-09	−0.0692	0.0573	0.2266
rs82334	4	C	A	0.3177	−0.0223	0.0035	3.13E-10	0.0063	0.0406	0.8761
rs9275160	6	A	G	0.3397	0.0382	0.0035	7.18E-28	0.0475	0.0443	0.2842

SNP, single-nucleotide polymorphisms; Chr, chromosome; EA, effect_allele; OA, other_allele; EAF, effect allele frequency.

**TABLE 2:** Instrumental variable test in the causal analysis of the FI and delirium.

Exposure variable	Number of instrumental variables	F value		MR-Egger regression		Leave-one-out sensitivity analysis
		**Max**	**Min**	**Intercept**	***P*-value**	***P*-value**
Frailty index	14	30	119.1	−0.011	0.832	0.058

### 3.2 Estimation results of the MR method

**FIGURE 1:**
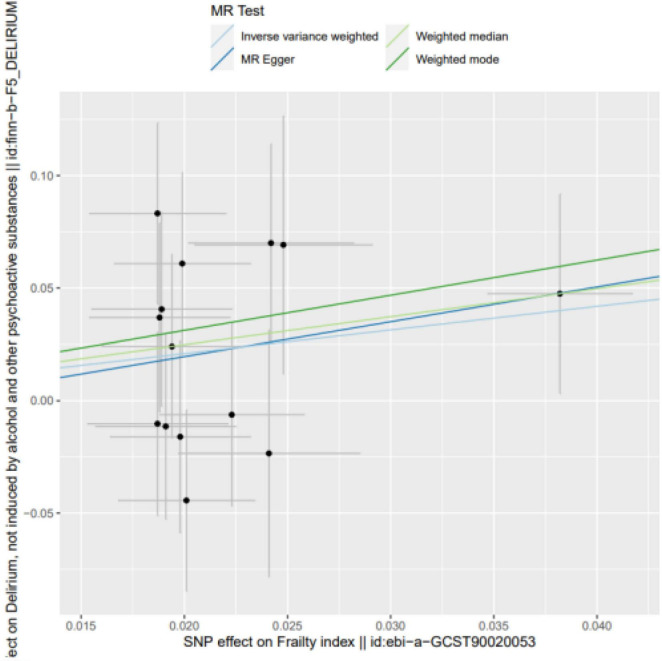
SNP effects on the outcome against SNP effects on the exposure. Frailty might increase the relative risk of delirium, as demonstrated by IVW (OR = 1.849, 95% CI 0.027∼2.067, *P* = 0.044), the weighted median (OR = 1.726, 95% CI –0.178∼2.664, *P* = 0.083) and MR-Egger regression (OR = 1.768, 95% CI –3.08∼6.171, *P* = 0.525).

**FIGURE 2:**
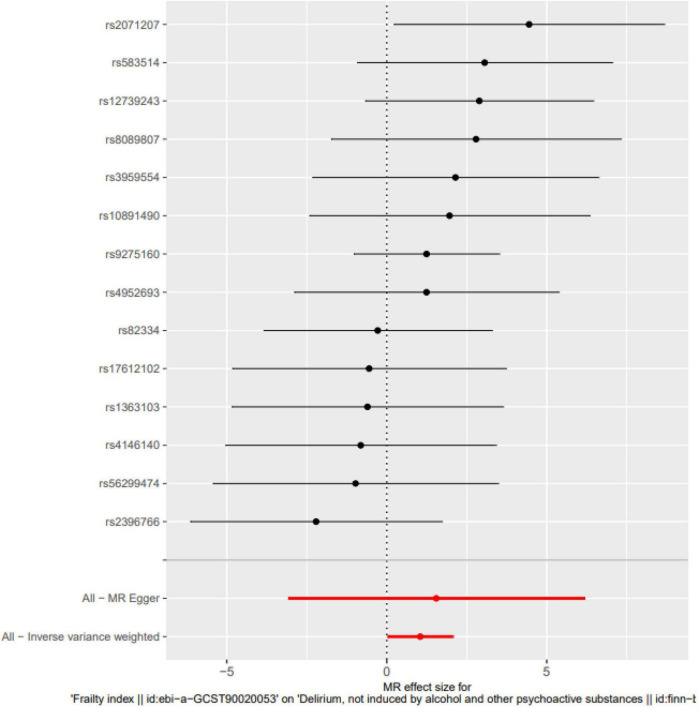
Single SNP analysis. The Single SNP analysis showed IVW (OR = 1.849, 95% CI 0.027∼2.067, *P* = 0.044), and MR-Egger regression (OR = 1.768, 95% CI –3.08∼6.171, *P* = 0.525).

Frailty might increase the relative risk of delirium, as demonstrated by IVW (OR = 1.849, 95% CI 0.027∼2.067, *P* = 0.044), the weighted median (OR = 1.726, 95% CI −0.178∼2.664, *P* = 0.083) and MR-Egger regression (OR = 1.768, 95% CI −3.08∼6.171, *P* = 0.525). Although the WME method and MR–Egger regression analysis showed no statistically significant causal relationship between the FI and the risk of delirium, the direction of the causal effect was consistent with the IVW method (see Figures 1, 2).

### 3.3 Horizontal pleiotropic and heterogeneity analysis

**FIGURE 3:**
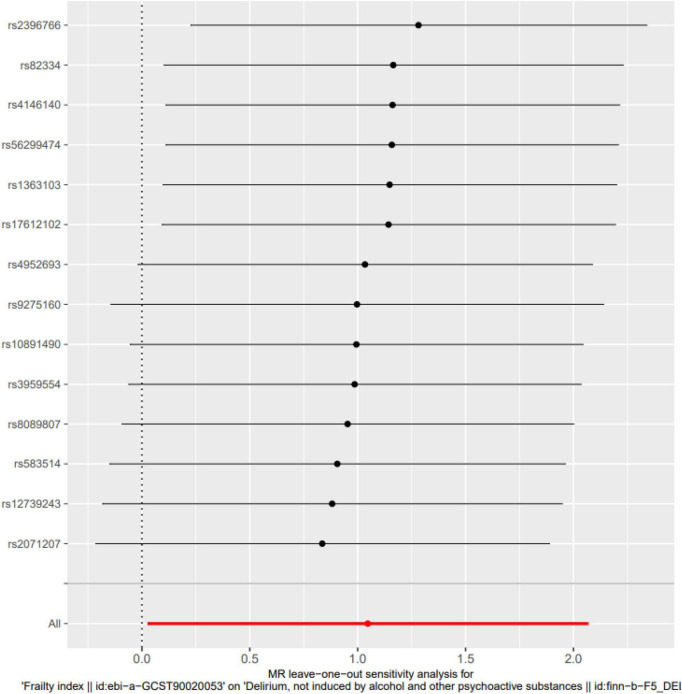
Leave-one-out sensitivity analysis. The “leave-one-out” sensitivity analysis showed that the included SNPs had no significant impact on the results (*P* = 0.058).

**FIGURE 4:**
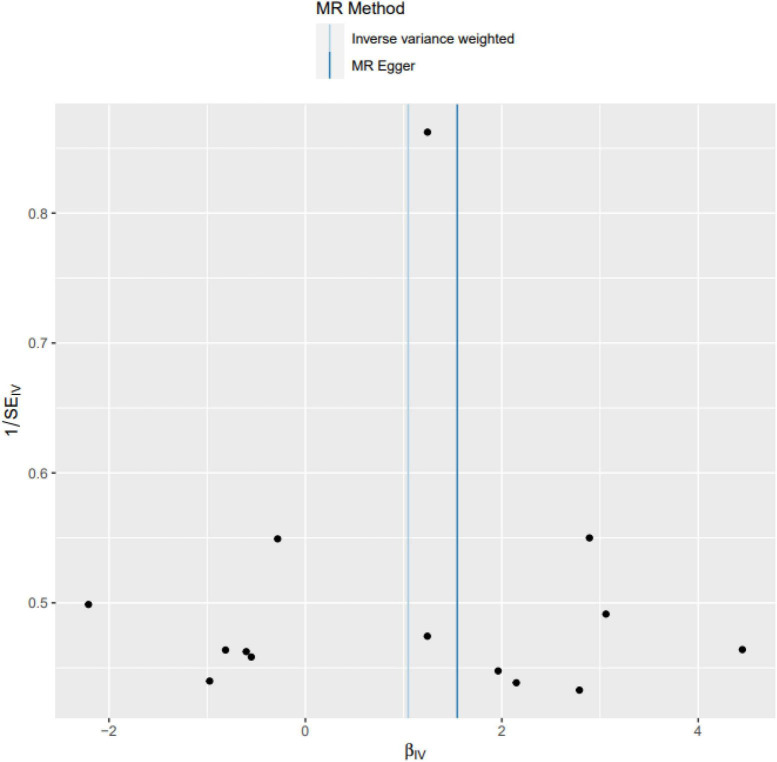
Funnel plot. The funnel plot also showed that the included SNPs had no significant impact on the results.

MR-Egger intercept analysis showed that there was no horizontal pleiotropic effect in this study (*P* = 0.832). Second, Cochran’s Q test showed that there was no certain heterogeneity in the study results (*P* = 0.583), and the “leave-one-out” sensitivity analysis and funnel plot also showed that the included SNPs had no significant impact on the results (see Figures 3, 4).

## 4 Discussion

This scientific investigation employed a Mendelian randomization analysis using a two-sample approach to evaluate the causality between the FI and the likelihood of experiencing delirium. The study uncovered a potential exacerbating impact of frailty on the risk of delirium in hospitalized patients, providing a basis for establishing a causal relationship between frailty and delirium. Delirium, characterized by impaired attention and confusion, represents a frequently encountered complication among hospitalized individuals, particularly among the elderly The occurrence of delirium can ascend to as high as 60%. A pharmacoepidemiological study was to describe the pattern of use of antidepressant medication the elderly population. Meantime, pharmacological treatment of delirium is receiving increasing attention (16). Concurrently, frailty is highly prevalent among elderly patients admitted to hospitals. Nonetheless, the presence of a causal association between these two phenomena remains ambiguous. The significance of the link between frailty and delirium warrants discussion. Despite the seemingly intuitive nature of this relationship, the current body of literature providing evidence for it is lacking, with most studies focusing on surgical contexts (17–19). And the relevant relationship between frailty and cardiovascular diseases and sarcopenia were also got the same results (20, 21). To shed light on this matter, Zhang et al. (6) executed a meta-analysis in 2021 exploring the interplay between frailty and delirium. The analysis divulged that the incidence of delirium in frail hospitalized patients was approximately three times higher compared to their non-frail counterparts (6). It is essential to consider, however, that the majority of studies included in the meta-analysis were of an observational nature, engendering a high level of heterogeneity among the included studies, thus limiting the ability to definitively establish a causal connection between frailty and delirium. A single-center study examined the impact of frailty and delirium on patient survival, revealing that frail individuals with delirium faced a higher long-term mortality risk compared to those who were fit (22). This finding aligns with the current study’s results and with previous research highlighting the significant influence of frailty on mortality (23, 24).

Our study possesses various strengths in contrast to observational studies. Firstly, we employed a comprehensive large-sample genome-wide association study, enabling a thorough analysis of delirium events. Secondly, we utilized several alternative methods that produced consistent results. However, it is crucial to exercise caution when interpreting these findings as certain limitations exist. Initially, the MR study employed GWAS data sourced exclusively from the European population, necessitating further studies to determine the generalizability of our research to other populations. Additionally, our study employed three statistical methods, with only the IVW method yielding statistically significant results. This may possibly be attributed to the fact that our study only included European population, leading to reduced statistical power in the causality assessment. Hence, it is imperative to conduct additional causality assessments encompassing diverse racial backgrounds in order to attain more reliable conclusions.

In conclusion, this study is the first to comprehensively explore the causal relationship between the FI and the risk of delirium in hospitalized patients through two-sample MR analysis. The results showed that there was a notable correlation between a higher FI and an elevated risk of delirium. This indicates that healthcare providers should take proactive measures to prevent delirium in hospitalized patients with a higher FI.

## Data availability statement

The datasets presented in this study can be found in online repositories. The names of the repository/repositories and accession number(s) can be found below: https://www.ebi.ac.uk/metagenomics/,ebi-a-GCST90020053.

## Ethics statement

Ethical review and approval was not required for the study on human participants in accordance with the local legislation and institutional requirements. Written informed consent from the patients/participants or patients/participants legal guardian/next of kin was not required to participate in this study in accordance with the national legislation and the institutional requirements.

## Author contributions

YC: Writing – original draft, Writing – review & editing. FF: Funding acquisition, Writing – review & editing. QL: Methodology, Resources, Writing – review & editing. HG: Software, Writing – review & editing. LZ: Software, Writing – review & editing. JL: Conceptualization, Supervision, Writing – review & editing.

## Supplementary material

Supplementary Table 1 The detail information of frailty datasets.

Supplementary Table 2 The detail basal information of delirium datasets.

Supplementary Figure 1 Survival analyses between endpoints of delirium datasets.

Supplementary Figure 2 Cumulative incidence of delirium.
