# Corrigendum: Frailty index and risk of delirium in hospitalized patients: a two-sample Mendelian randomization study

**DOI:** 10.3389/fmed.2024.1485061

**Published:** 2024-09-16

**Authors:** Yu Chen, Fang Feng, Qun Li, Hong Guo, Lu Zhang, Jian Liu

**Affiliations:** ^1^The First School of Clinical Medicine of Lanzhou University, Lanzhou, Gansu, China; ^2^Intensive Care Unit, Lanzhou University Second Hospital, Lanzhou, Gansu, China; ^3^Intensive Care Unit, The First School of Clinical Medicine of Lanzhou University, Lanzhou, Gansu, China; ^4^Intensive Care Unit, Gansu Provincial Central Hospital, Lanzhou, Gansu, China

**Keywords:** frailty index, delirium, Mendelian randomization study, intensive care, nursing care

In the published article, there was an error in affiliation(s) [1]. Instead of “Intensive Care Unit, The First Hospital of Lanzhou University, Lanzhou, Gansu, China,” it should be “The First School of Clinical Medicine of Lanzhou University, Lanzhou, Gansu, China.”

The authors apologize for this error and state that this does not change the scientific conclusions of the article in any way. The original article has been updated.

