# Supplementary figures and images for: Frailty index and risk of delirium in hospitalized patients: a two-sample Mendelian randomization study
Source: Front Med (Lausanne). 2024 May 22;11:1361437. doi: 10.3389/fmed.2024.1361437 (PMC11150602; doi:10.3389/fmed.2024.1361437)

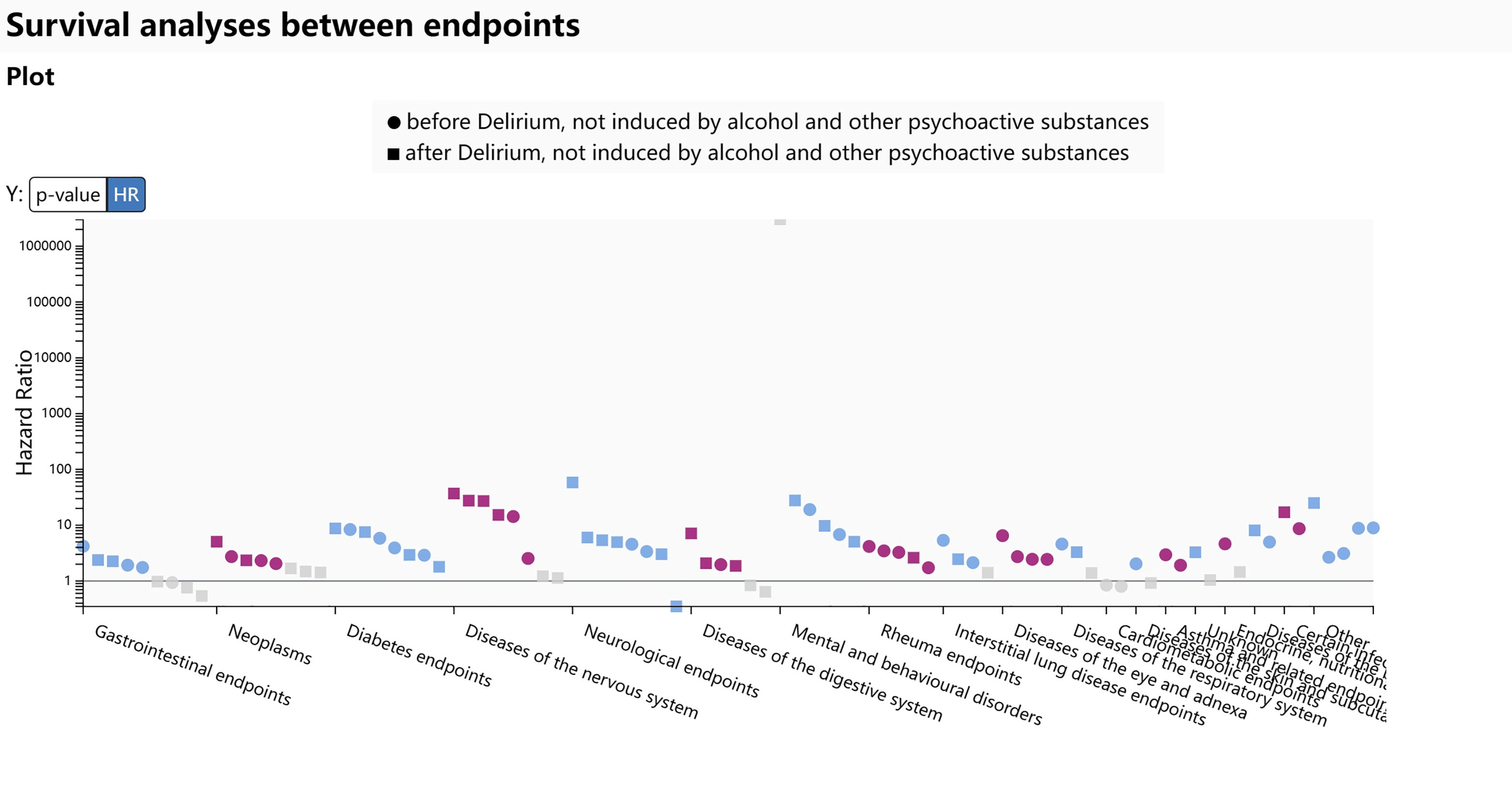

Supplement: Supplementary Figure 1 — Survival analyses between endpoints of delirium datasets. [file Image_1.PNG]

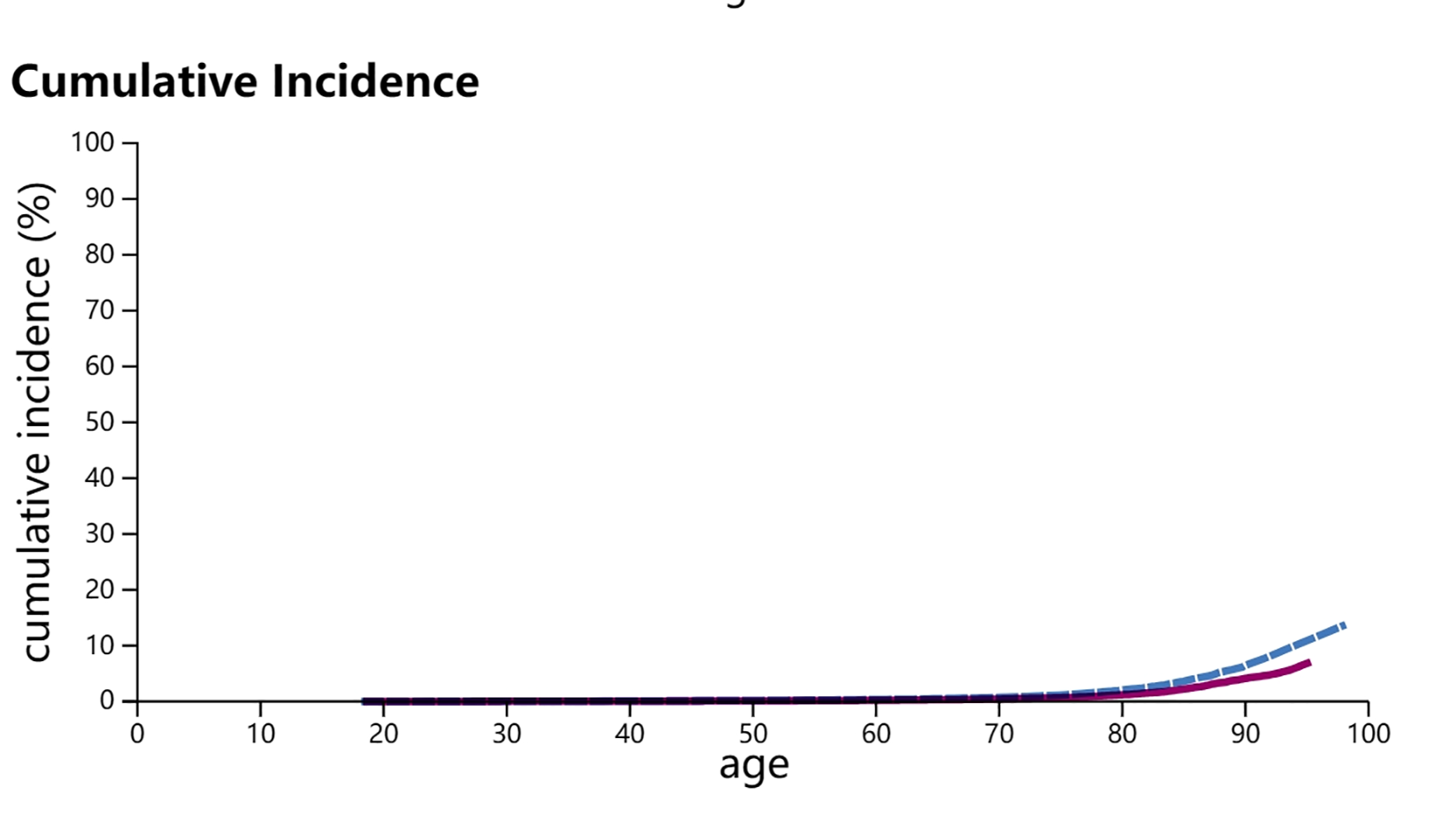

Supplement: Supplementary Figure 2 — Cumulative incidence of delirium. [file Image_2.PNG]
